# Thirteen weeks of supplementation of vitamin D and leucine-enriched whey protein nutritional supplement attenuates chronic low-grade inflammation in sarcopenic older adults: the PROVIDE study

**DOI:** 10.1007/s40520-019-01208-4

**Published:** 2019-05-02

**Authors:** Keliane Liberman, Rose Njemini, Yvette Luiking, Louis N. Forti, Sjors Verlaan, Jürgen M. Bauer, Robert Memelink, Kirsten Brandt, Lorenzo M. Donini, Marcello Maggio, Tony Mets, Sander L. J. Wijers, Cornel Sieber, Tommy Cederholm, Ivan Bautmans

**Affiliations:** 1 Frailty in Ageing Research Group (FRIA), Vrije Universiteit Brussel (VUB), Laarbeeklaan 103, 1090 Brussels, Belgium; 2 Nutricia Research, Nutricia Advanced Medical Nutrition, Utrecht, The Netherlands; 3 Department of Internal Medicine, Section of Gerontology and Geriatrics, VU University Medical Center, Amsterdam, The Netherlands; 4 Center of Geriatric Medicine, Heidelberg University, Heidelberg, Germany; 5 Faculty of Sports and Nutrition, Amsterdam University of Applied Sciences, Amsterdam, The Netherlands; 6 Human Nutrition Research Centre, Institute of Cellular Medicine, Newcastle University, Newcastle upon Tyne, UK; 7 Department of Experimental Medicine, Section of Medical Pathophysiology, Endocrinology and Human Nutrition, “Sapienza” University of Rome, Rome, Italy; 8 Department of Medicine and Surgery, Section of Geriatrics, University of Parma, Parma, Italy; 9 Friedrich-Alexander-Universität Erlangen-Nürnberg, Erlangen, Germany; 10 Department of Public Health and Caring Nutrition Sciences/Clinical and Metabolism, Department of Geriatric Medicine, Uppsala University Hospital, Uppsala, Sweden

**Keywords:** Vitamin D, Leucine, Whey proteins, Dietary supplements, Cytokines, Aged

## Abstract

**Background:**

A chronic low-grade inflammatory profile (CLIP) is associated with sarcopenia in older adults. Protein and Vitamin (Vit)D have immune-modulatory potential, but evidence for effects of nutritional supplementation on CLIP is limited.

**Aim:**

To investigate whether 13 weeks of nutritional supplementation of VitD and leucine-enriched whey protein affected CLIP in subjects enrolled in the PROVIDE-study, as a secondary analysis.

**Methods:**

Sarcopenic adults (low skeletal muscle mass) aged ≥ 65 years with mobility limitations (Short Physical Performance Battery 4–9) and a body mass index of 20–30 kg/m^2^ were randomly allocated to two daily servings of active (*n* = 137, including 20 g of whey protein, 3 g of leucine and 800 IU VitD) or isocaloric control product (*n* = 151) for a double-blind period of 13 weeks. At baseline and after 13 weeks, circulating interleukin (IL)-8, IL-1 receptor antagonist (RA), soluble tumor-necrosis-factor receptor (sTNFR)1, IL-6, high-sensitivity C-reactive protein, pre-albumin and 25-hydroxyvitamin(OH)D were measured. Data-analysis included repeated measures analysis of covariance (corrected for dietary VitD intake) and linear regression.

**Results:**

IL-6 and IL-1Ra serum levels showed overall increases after 13 weeks (*p* = 0.006 and *p* < 0.001, respectively). For IL-6 a significant time × treatment interaction (*p* = 0.046) was observed, with no significant change over time in the active group (*p* = 0.155) compared to control (significant increase *p* = 0.012). IL-8 showed an overall significant decrease (*p* = 0.03). The change in pre-albumin was a significant predictor for changes in IL-6 after 13 weeks.

**Conclusions:**

We conclude that 13 weeks of nutritional supplementation with VitD and leucine-enriched whey protein may attenuate the progression of CLIP in older sarcopenic persons with mobility limitations.

**Electronic supplementary material:**

The online version of this article (10.1007/s40520-019-01208-4) contains supplementary material, which is available to authorized users.

## Introduction

Ageing is accompanied with a chronic low-grade inflammatory profile (CLIP) reflected by subtle increases in circulating cytokines [1, 2]. CLIP has been associated with frailty in older adults [3, 4], increasing vulnerability to poor health outcomes such as disability, hospital admission and mortality [5]. Sarcopenia, a contributor to physical frailty, is a muscle failure disease that is caused by adverse muscle changes that accumulate over life [6]. A blunted response of muscle protein synthesis to nutrient intake is one of the greatest limitations to muscle preservation, which may be induced by inflammation among other factors [7].

Leucine, a branched-chain amino acid, can induce both anti-inflammatory and pro-inflammatory effects, probably depending on its circulating concentration [8–12]. Previous studies showed that the recommended amount of protein intake for older adults should be 1.0–1.5 g of proteins per kilogram body weight per day [13]. However, a majority of older adults does not reach these amounts of dietary protein intake. Circulating vitamin (Vit)D is inversely related to IL-6 and CRP, and has an anti-inflammatory effect by contributing to the regulation of immune cells [14, 15]. VitD deficiency is often present in older adults due to decreased UV-light exposure and reduced VitD synthesis and metabolism [16]. Therefore, VitD supplementation is often indicated [17].

Studies investigating the anti-inflammatory effect of nutritional supplementation show contradicting results [18–20]. In the PROVIDE study, 13 weeks of VitD and leucine-enriched whey protein supplementation improved muscle mass and lower extremity function among sarcopenic older adults [21]. In addition, this study showed that sarcopenic participants with higher baseline circulating VitD concentrations and higher dietary protein intake obtained greater gains in muscle mass after 13 weeks intervention [22]. In the present sub-study, we investigated whether 13 weeks of nutritional supplementation affected circulating inflammatory markers in older sarcopenic adults enrolled in the PROVIDE study.

## Materials and methods

### Participants

**Fig. 1:**
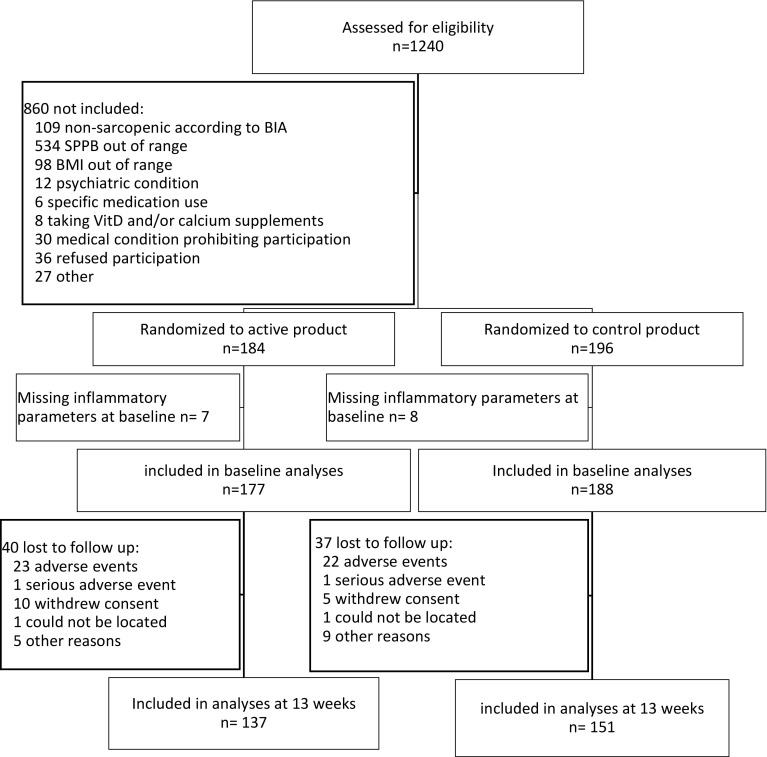
Flow chart

Detailed information on the PROVIDE study protocol was published previously [21] and can be found on https://www.trialregister.nl/trialreg with identifier: NTR2329. Subjects aged ≥ 65 years, with mild to moderate limitations in physical functioning (Short Physical Performance Battery (SPPB) score 4–9), with class I or II sarcopenia (skeletal muscle mass/BW × 100) < 37% in men and < 28% in women using bio-impedance analyses) [23], a body mass index (BMI) of 20–30 kg/m^2^ and providing written informed consent were eligible for participation [21]. Eligible candidates were evaluated for in -and exclusion criteria during a screening visit as described elsewhere [21]. Briefly, 1240 participants from 6 European countries (Belgium, Germany, Ireland, Italy, Sweden and the United Kingdom) were assessed for eligibility. 380 were randomly allocated by Danone Nutricia Research to either the active (*n* = 184) or the control group (*n* = 196) via permuted block randomization (block size 4) stratified for SPPB categories 4–6 and 7–9 and study center. The randomization sequence was computer-generated by a blinded statistician not involved in data collection or analysis. All investigators, study staff, and participants were blinded to group allocations (see Ref. [21] for details). 297 completed the 13 weeks intervention. In total, 78 from the 380 randomized subjects terminated the RCT study early (38 in control and 40 in active group). From the 78 early terminators, 45 subjects did not continue with the study because of an adverse event (AE), 2 subjects did not continue because of serious AE (assessed as not related to the study product), 15 subjects withdrew their informed consent, 2 subjects were lost to follow-up, in 1 subject a protocol deviation occurred (the subject took calcium supplements) leading to study discontinuation and 13 subjects had another reason for discontinuation. This sub-study is a secondary analysis based on subjects of whom inflammatory biomarkers were available (Fig. 1).

### Intervention

Subjects either received the active product or the control product for a double-blind period of 13 weeks, as two 40-g sachets to be dissolved in 125 ml of water and consumed before breakfast and lunch, respectively. Per serving, the active product contained 20 g of whey protein, 3 g total leucine, and a mixture of carbohydrates and fat providing 150 kcal per serving, 800 IU VitD and a mixture of fibres, minerals and vitamins. The isocaloric control product did not contain any protein or micronutrients; detailed compositions are shown in the Supplemental Table of the main PROVIDE publication [21].

### Outcome measures

At the baseline visit (within 1 week after the screening visit), characteristics such as age, sex, BMI, ethnicity, living situation, medical history, cognitive function (mini-mental state examination) [24] and pre-existing conditions, use of nutritional supplements and medication were recorded. At baseline and after 7 and 13 weeks, subjects underwent assessments including handgrip strength, body composition, physical performance test and activity, dietary intake and blood sampling as described previously [21]. The European version of the Physical Activity Scale for the Elderly (PASE) was used to assess physical activity. Body composition was measured using dual energy X-ray absorptiometry (DXA, different models from Hologic, Bedford, USA; and Lunar, Fairfield, USA). Dietary VitD and protein intake were assessed by a 3-day dietary record, including two week days and one weekend day. Dietary records were checked for completeness with participants during study visits and additional information was obtained about unclear items or amounts. Total energy, macronutrient and micronutrient intakes were calculated by the participating sites using country-specific dietary data entry systems and food composition tables.

After an overnight fast, serum samples were collected and frozen at – 80 °C until assayed for cytokines levels. IL-8 (ultrasensitive), IL-1RA and sTNFR1 were measured separately using commercially available ELISA kits (Lifetech, Carlsbad, CA), and IL-6 using an ultrasensitive singleplex bead kit (Lifetech, USA) as described previously [25]. Sensitivity levels were < 0.1 pg/ml (IL-8), 4 pg/ml (IL-1RA), 50 pg/ml (sTNFR1) and < 0.05 pg/ml (IL-6). For each participant the samples of both time points were analysed on the same plate to limit inter-assay variability. At baseline and 13 weeks, samples for determination of cytokines were available from respectively 365 and 288 subjects (Fig. 1). Baseline characteristics of subjects with missing data for cytokines were similar to those who were included in the analyses, except for female subjects with missing data in whom the PASE was significantly lower (47.61 ± 31.36 versus 99.96 ± 67.21, *p* < 0.001).

At baseline, and after 7 and 13 weeks, CRP, pre-albumin and 25-hydroxyvitamin(OH)D were determined by the central PROVIDE laboratory as described elsewhere [21, 22]. For these analyses, several samples were missing at baseline (*n* = 3 out of 365), 7 weeks (*n* = 10 out of 288) and 13 weeks (*n* = 17 out of 288). For 31 (out of 288) participants, no information was available for VitD intake throughout the study.

### Statistical analyses

Statistical analyses were performed in IBM SPSS v25.0.0.0 (SPSS Inc, Illinois, USA). Because of non-normal distribution (Kolmogorov–Smirnov Goodness of Fit test *p* < 0.05), all inflammatory markers were log (10)-transformed to reduce skewness and back transformed for data presentation (Supplementary Table 1). Baseline between group differences were analysed by unpaired *t* tests. Pearson correlations were computed between baseline inflammatory markers and other baseline outcomes.

Changes in cytokines over time were analysed with repeated-measures ANCOVA using time as within subject’s factor and intervention (active versus control product) group as between subject’s factor. Since dietary intake of VitD and protein might interfere with the nutritional supplement, the mean dietary intake of these components (average of intake measured at baseline, 7 weeks and 13 weeks) were entered in the models as covariates. We also verified whether other relevant factors including fat mass, NSAID use, SPPB, PASE, sex, and baseline 25(OH)D were significant covariates in the analyses. Since only mean dietary VitD intake was a significant factor, this was finally retained as covariate. In addition, these analyses were repeated including only participants with CRP ≤ 10 mg/Llthroughout the entire study to eliminate potential bias due to acute inflammatory conditions (reflected by CRP-value > 10 mg/l [26]).

Next, linear regression was used to appraise the proportional contribution of changes in circulating 25(OH)D and protein (reflected by circulating pre-albumin [27]) to the changes in those cytokines for which a significant time × treatment interaction was found. The change in cytokine level was used as dependent variable and mean dietary intake of VitD and protein, as well as change in pre-albumin and circulating 25(OH)D values were used as predictors. Significance was set at *p* < 0.05.

## Results

There were no significant differences between both groups at baseline (Table 1). Sex differences in body composition and muscle strength were reported previously [21] and were in line with the expectations.

**Table 1:** Participants’ baseline characteristics

	Active					Control					Between groups *p* value°
	Male		Female		*p* value°	Male		Female		*p* value°	
	*n*	Mean ± SD	*n*	Mean ± SD		*n*	Mean ± SD	*n*	Mean ± SD		
Age (years)	62	77.87 ± 6.60	115	77.17 ± 6.66	0.50	66	78.02 ± 7.45	122	78.00 ± 6.70	0.99	0.40
MMSE (score 0–30)	62	27.94 ± 2.34	113	28.58 ± 1.43	0.03	66	27.94 ± 3.18	121	28.63 ± 1.41	0.04	0.86
BMI (kg/m^2^)	62	26.27 ± 2.29	115	25.85 ± 2.69	0.30	66	26.73 ± 2.72	122	25.95 ± 2.79	0.07	0.42
Handgrip strength (kg)	62	25.81 ± 7.40	114	16.53 ± 5.70	< 0.01	66	26.17 ± 7.18	121	16.54 ± 5.29	< 0.01	0.87
SPPB total score (0–9)	62	7.77 ± 1.94	115	7.33 ± 1.90	0.14	66	7.61 ± 1.92	122	7.44 ± 1.90	0.58	0.94
PASE (score 0–793)	62	93.74 ± 64.59	113	107.70 ± 75.06	0.22	66	113.74 ± 94.24	118	92.56 ± 58.07	0.06	0.73
Fat mass (%)	50	30.99 ± 5.31	98	39.53 ± 5.03	< 0.01	53	31.34 ± 4.43	111	39.45 ± 4.92	< 0.01	0.76
ALM (kg)	58	21.99 ± 3.16	105	15.72 ± 2.70	< 0.01	58	21.44 ± 2.89	115	15.51 ± 2.30	< 0.01	0.29
NSAID use (*n*)	6		4		0.10^◊^	2		4		1.00^◊^	0.31^◊^
25(OH)D (nmol/l)	62	53.22 ± 24.22	115	49.83 ± 22.27	0.35	63	51.76 ± 19.02	122	50.81 ± 22.96	0.78	0.96
Dietary VitD intake (µg/day)	52	3.38 ± 3.14	103	2.93 ± 2.85	0.37	52	2.89 ± 5.56	110	3.79 ± 4.21	0.26	0.35
Dietary protein intake (g/kg body weight/day)	62	1.00 ± 0.28	108	1.06 ± 0.31	0.17	65	0.96 ± 0.23	116	1.03 ± 0.31	0.12	0.26
Pre-albumin (g/l)	62	0.27 ± 0.07	115	0.25 ± 0.04	0.19	63	0.27 ± 0.048	122	0.26 ± 0.06	0.14	0.53
Log_sTNFR1 (ng/ml)	62	0.43 ± 0.13	115	0.44 ± 0.13	0.44	66	0.48 ± 0.15	122	0.45 ± 0.15	0.16	0.08
Log_IL8 (pg/ml)	62	0.61 ± 0.26	115	0.56 ± 0.28	0.32	66	0.64 ± 0.30	122	0.61 ± 0.27	0.52	0.15
Log_IL1RA (pg/ml)	62	1.88 ± 0.48	115	1.98 ± 0.37	0.12	66	1.95 ± 0.39	122	1.95 ± 0.45	0.96	0.97
Log_IL-6 (pg/ml)	62	0.29 ± 0.37	115	0.26 ± 0.46	0.65	66	0.29 ± 0.48	122	0.28 ± 0.44	0.75	0.85
Log_CRP (mg/l)	62	0.40 ± 0.53	115	0.26 ± 0.43	0.05	63	0.25 ± 0.50	122	0.31 ± 0.46	0.41	0.68

*BMI* body mass index (weight/height^2^), *SPPB* Short Physical Performance Battery, *PASE* Physical Activity Scale for the Elderly, *ALM* appendicular lean mass, *NSAID* non-steroidal anti-inflammatory drugs

°Unpaired *t* test

^◊^Fischer exact test

As shown in Table 2, baseline cytokines and dietary protein intake were not significantly correlated. Higher dietary VitD intake and circulatory 25(OH)D were significantly related to lower IL-8. Baseline SPPB and PASE were negatively correlated with baseline cytokines. Fat mass correlated negatively with IL-8 and positively with CRP.

**Table 2:** Associations between baseline inflammatory parameters, and vitamin D and protein status and intake, functional status, and fat mass

	VitD intake (µg/day)	25(OH)D (nmol/l)	Protein intake (g/kg body weight)	Pre-albumin (g/l)	SPPB (score 0–9)	Fat mass (kg)	PASE (score 0–793)
Log_sTNFR1 (ng/ml)	− 0.09	− 0.07	0.02	− 0.065	− 0.27**	− 0.05	− 0.25**
Log_IL8 (pg/ml)	− 0.19**	− 0.12*	0.05	− 0.136**	− 0.20**	− 0.19**	− 0.16**
Log_IL1RA (pg/ml)	0.07	− 0.06	− 0.07	− 0.083	− 0.10	0.07	− 0.11*
Log_IL6 (pg/ml)	− 0.04	− 0.09	0.04	− 0.173**	− 0.14**	0.01	− 0.15**
Log_CRP (mg/l)	− 0.08	− 0.05	− 0.07	− 0.166**	− 0.14**	0.18**	− 0.13*

Pearson correlation coefficients **p < 0.01, * *p* < 0.05

**Fig. 2:**
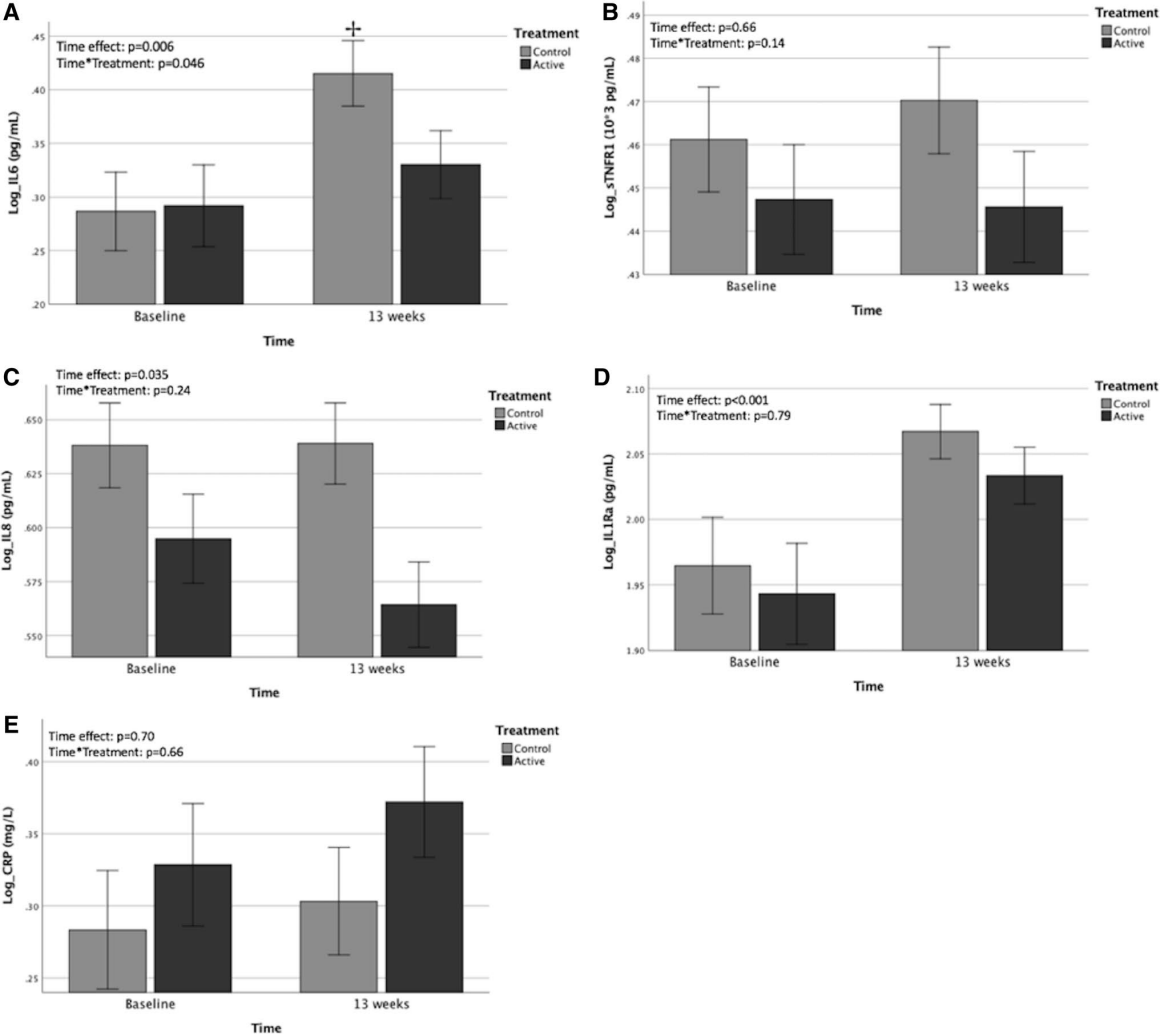
Effects of active versus control intervention on inflammatory markers. **a** IL-6, **b** sTNFR1, **c** IL-8, **d** IL1Ra, **e** CRP bars represent mean values ± 1SD corrected for mean dietary VitD intake as a covariate. Repeated measures ANCOVA; ^†^significantly different from baseline *p* < 0.05

IL-6 and IL-1Ra showed an overall significant increase after 13 weeks (*p* = 0.006 and *p* < 0.001, respectively; Fig. 2). For IL-6 a significant time × treatment interaction (*p* = 0.046) showed that the increase was attenuated in the active (no increase: 1.95 ± 1.09 to 2.17 ± 1.08 pg/ml; *p* = 0.155) compared to control (significant increase: 1.96 ± 1.09 to 2.56 ± 1.07 pg/ml; *p* = 0.012) group. IL-8 showed an overall significant decrease (*p* = 0.03), but there was no significant time × treatment interaction (*p* = 0.24). Back transformed data on changes over time can be found in Supplementary Table 1.

To eliminate potential bias due to acute inflammatory conditions during the study, we identified all participants who showed a CRP-value > 10 mg/l—as an indicator for an active inflammatory condition [26]—at any time point during the study. In participants with baseline CRP ≤ 10 mg/l, 14 persons acquired an inflammatory profile (i.e. CRP-value > 10 mg/l) after 7 weeks intervention (10 in the active and 4 in the control group, *p* = 0.057). At 13 weeks, three participants retained an inflammatory profile (two in the active and one in the control group, *p* = 0.913). For participants showing no inflammation in the previous period, 13 acquired an inflammatory profile at week 13 (6 in the active and 7 in the control group, *p* = 0.956). When including only participants with CRP ≤ 10 mg/l throughout the entire study (*n* = 227; active *n* = 103 and control *n* = 124; subjects with missing data for CRP were also excluded), comparable results, though more pronounced, were found (see Supplementary Fig. 1).

Finally, we computed a linear regression model to appraise the proportional association of changes in circulating 25(OH)D and pre-albumin to the changes in IL-6 (Table 3). Only the change in pre-albumin was significantly associated with changes in IL-6 after 13 weeks.

**Table 3:** Explanatory regression analysis for changes in IL-6 after 13-weeks

*R*² = 0.049, *p* = 0.015	*B* coefficients	*p* value	95.0% Confidence interval for *B*	
			Lower bound	Upper bound
Change in Log_IL6				
(Constant)	0.114	0.099	− 0.022	0.250
Dietary VitD intake	− 0.002	0.812	− 0.016	0.013
Dietary protein intake	< 0.001	0.830	− 0.002	0.002
Change in circulating pre-albumin	− 1.685	0.002	− 2.766	− 0.604
Change in circulating 25(OH)D	− 0.002	0.105	− 0.004	< 0.001

A multiple linear regression model was computed with change in Log_IL6 as dependent variable, and mean dietary intake of VitD and protein, as well as change in pre-albumin and circulating 25(OH)D values as predictors. Only the change in circulating pre-albumin was significantly associated with changes in Log_IL-6 after 13 weeks; in fact, higher increase in circulating pre-albumin was associated with lower changes in Log_IL6 (*B* coefficient = − 1.685, *p* = 0.002), independently from the other factors entered in the model

## Discussion

We assessed the effect of a 13-week VitD and leucine-enriched whey protein oral nutritional intervention on CLIP in sarcopenic older adults with mobility limitations. We found an overall increase in CLIP (demonstrated by IL-6 and IL-1RA), which was significantly attenuated in the active group compared to control for IL-6. When excluding participants who might have experienced pathologic acute inflammation during the study (based on CRP-values > 10 mg/l), the anti-inflammatory effects of the active intervention remained significant, and was even more pronounced.

CLIP is a well-known phenomenon in the aged [1, 28–30]. Contributing factors include immunosenescence, lack of physical activity, decline of sex hormones and increase in adipose tissue [2, 31–34]. The exact kinetics of CLIP are not well described in the literature. However, CLIP is more pronounced in (pre)frail older adults and/or older subjects with chronic diseases [1, 4, 35–37]. Given the specific profile of our participants with low muscle mass and mobility limitations, it can be expected that CLIP would have progressed more rapidly than expected in a group of healthier older persons. The IL-6 levels in our participants (median = 1.97 pg/ml, P25–P75 = [1.19–2.96]) correspond to the levels for CLIP (2.13 pg/ml [1.37–4.23]) as recently reviewed [30]. The cross-sectional data reviewed by Calder et al. [30] suggest mean differences of about 0.7 pg/ml for log IL-6 between young and older adults. In this context the difference in change in IL-6 between active and control group that we observed might be of clinical relevance.

Our findings are in line with a recently published RCT investigating the additional effect of a combined VitD and whey protein oral nutritional supplement on exercise-induced changes in CRP in sarcopenic older persons [38]. They reported a significant time × treatment effect after 12 weeks intervention (*p* = 0.04) characterized by a tendency for CRP to increase in the placebo group (+ 4.4 mg/l, *p* = 0.06) which was attenuated in the intervention group (− 1.9 mg/l, *p* = 0.33) [38]. However, these authors did not quantify other biomarkers of CLIP.

VitD and protein supplementation can reduce CLIP through several pathways. VitD inhibits T-cell proliferation, inhibiting Th1 and Th17 pro-inflammatory responses and stimulating Th2 response, resulting in a decreased inflammatory profile [15, 39]. Inflammatory cells can convert VitD into calcitriol, which can itself regulate the cytokines by blocking NF-kß p65 activation (by upregulation of ikBa) which in its turn can inhibit differentiation of B-cells to plasma cells [40]. In dendritic cells, VitD3 downregulates the expression of pro-inflammatory cytokines, inhibits differentiation of plasma cells and upregulates expression of anti-inflammatory cytokines (such as IL-10) as well as inflammation-inhibiting molecules such as ILT-3 [41]. Dietary proteins are crucial for muscle anabolism and considered to play a major role in countering sarcopenia and CLIP [42]. Amino acids such as leucine have strong anabolic effects and stimulate intramuscular protein synthesis via upregulation of the mTOR pathway, which also reduces protein breakdown and can induce anti-inflammatory effects [8, 43]. Also β-hydroxy β-methylbutyrate, a metabolite of leucine, has anti-inflammatory effects in older persons [44].

As both VitD and protein—as main components of the nutritional supplement—can have anti-inflammatory effects, linear regression analysis was used to appraise the proportional contribution of changes in circulating 25(OH)D and protein (reflected by circulating pre-albumin) to the changes in IL-6. Our results point towards the effects of the protein component of the nutritional supplementation (reflected by the changes in circulating pre-albumin) as significantly associated with the attenuation in IL-6 increase. Although many studies investigated the effect of nutritional supplementation on physical outcomes, very few investigated the effects on inflammation in older adults [45, 46]. Most studies did not find any effect of VitD on inflammation [47, 48]. Studies reporting significant effects were contradictory and remained inconclusive (see Ref. [49] for review).

Our findings regarding IL-8 are in line with those reported by Yusupov et al. who found that IL-8 decreased significantly after 12 weeks of VitD supplementation (− 48%, *p* < 0.001), which was comparable to the decrease in the control group (− 33%, *p* = 0.02) [50]. Nakashyan et al. recently performed a study on constitutive and IL-1ß-stimulated human gingival fibroblast, where they analysed the effect of VitD on levels of IL-6. In constitutive fibroblast, IL-6 production decreased by ~ 50%. When cells were stimulated with IL-1ß, the effects on IL-6 depended on the time when the cells were exposed to VitD. The longer the time in between the IL-1ß stimulus and the VitD exposure, the smaller the effect observed [51]. Considering the inflammatory effects of IL-1ß, this is comparable to the results obtained in our analyses, where a time effect was seen when all participants were included. When participants who acquired an inflammatory profile (i.e. CRP > 10 mg/l) were excluded from our analyses, the effects on IL-6 were more pronounced. In our study, the levels of circulating sTNFR1 were not related to protein or VitD, and did not show changes over time. Similarly, Vita et al. found no correlation between VitD and sTNFR1 or IL-1Ra in older adults [14].

The strengths of this study are the double-blinded randomized and controlled design and the large sample size. Also, the analyses with high-sensitivity ELISA kits allowed to detect small changes in inflammatory markers. However, the results should be interpreted cautiously, since the nutritional supplement contained other compounds besides VitD, leucine and whey proteins which might have influenced the anti-inflammatory effects. As reported earlier [21, 22], the active product contained micronutrients, including also 2.2 g zinc per serving whereas the iso-caloric control product contained only carbohydrates and fat. Dietary zinc intake as well as circulating zinc levels are inversely related to IL-6, TNF-α and CRP [52]. In addition, Bao et al. found that IL-6 decreased significantly after 6 months of zinc supplementation in 40 older adults [53]. Unfortunately, we have no data regarding zinc status in our participants. Another potential limitation is that the dose of the oral nutritional supplement might not have been sufficiently high to obtain optimal effects. In the study performed by Azizieh et al. participants with deficient VitD levels and high CRP showed higher levels of pro-inflammatory cytokines compared to participants with sufficient VitD levels [54]. In our study, the supplementation dose was not tailored according to, e.g. baseline 25(OH)D status or nutritional protein intake. On the other hand, we used dietary VitD intake as a covariable in our analyses, and the influence of other potential confounders such as dietary protein intake, physical functioning, physical activity and NSAID use has been verified. In this study we used pre-albumin level as a surrogate marker for the protein-related nutritional status. However, since pre-albumin level can—at least in acute inflammatory conditions—be influenced by ongoing inflammation [55, 56], we cannot exclude that this might have affected our results. Therefore, the role of the proteins in the supplement on the attenuation of CLIP in our study needs to be interpreted with caution. 25% of the randomized subjects were lost to follow-up or showed missing data, which is relatively high but acceptable for a clinical trial in older patients with mobility limitations. Finally, as this was a multi-center study, environmental factors such as season of the year or exposure to sunlight—which is a source of VitD—might have influenced the results [57].

## Conclusions

Based on the results of our study, we conclude that 13 weeks of nutritional supplementation with VitD and leucine-enriched whey protein may attenuate the progression of chronic low-grade inflammation in older sarcopenic persons with mobility limitations. Our results can be of clinical significance since chronic low-grade inflammation is a major contributor to the progression of frailty and sarcopenia.

## Electronic supplementary material

Below is the link to the electronic supplementary material.

Supplementary material 1 (DOCX 271 kb)
