# Supplementary material for: Thirteen weeks of supplementation of vitamin D and leucine-enriched whey protein nutritional supplement attenuates chronic low-grade inflammation in sarcopenic older adults: the PROVIDE study
Source: Aging Clin Exp Res. 2019 May 2;31(6):845–54. doi: 10.1007/s40520-019-01208-4 (PMC6583678; doi:10.1007/s40520-019-01208-4)
Supplement: Supplementary file 1 — Supplementary material 1 (DOCX 271 kb) [file 40520_2019_1208_MOESM1_ESM.docx]

**SUPPLEMENTARY MATERIALS**

|  |  | **ACTIVE** | | | | **CONTROL** | | | |
| --- | --- | --- | --- | --- | --- | --- | --- | --- | --- |
|  |  |  |  | **95.0% CI** | |  |  | **95.0% CI** | |
|  |  | **Mean** | **std** | **low** | **up** | **Mean** | **std** | **low** | **up** |
| **IL-6 (pg/ml)** | **T0** | 1.95 | 1.09 | 1.66 | 2.31 | 1.96 | 1.09 | 1.66 | 2.31 |
|  | **T2** | 2.17 | 1.08 | 1.86 | 2.54 | 2.56 | 1.07 | 2.24 | 2.93 |
| **sTNFR1 (ng/ml)** | **T0** | 2.82 | 1.03 | 2.62 | 2.98 | 2.90 | 1.03 | 2.72 | 3.08 |
|  | **T2** | 2.82 | 1.03 | 2.66 | 2.99 | 2.96 | 1.03 | 2.79 | 3.13 |
| **IL-8 (pg/ml)** | **T0** | 3.97 | 1.05 | 3.61 | 4.36 | 4.33 | 1.05 | 3.94 | 4.73 |
|  | **T2** | 3.69 | 1.04 | 3.37 | 4.03 | 4.36 | 1.04 | 3.98 | 4.75 |
| **IL-1Ra (pg/ml)** | **T0** | 87.10 | 1.10 | 72.61 | 104.71 | 92.68 | 1.09 | 78.89 | 109.14 |
|  | **T2** | 107.65 | 1.06 | 96.61 | 120.23 | 116.95 | 1.04 | 107.40 | 127.35 |
| **CRP (mg/l)** | **T0** | 2.12 | 1.11 | 1.72 | 2.61 | 1.86 | 1.10 | 1.54 | 2.24 |
|  | **T2** | 2.44 | 1.09 | 2.06 | 2.90 | 2.01 | 1.10 | 1.67 | 2.42 |

**Supplementary table 1. Back transformed data of the estimated mean marginals of the ANCOVA of the inflammatory cytokines.** Mean vitamin D intake was inserted as covariable.


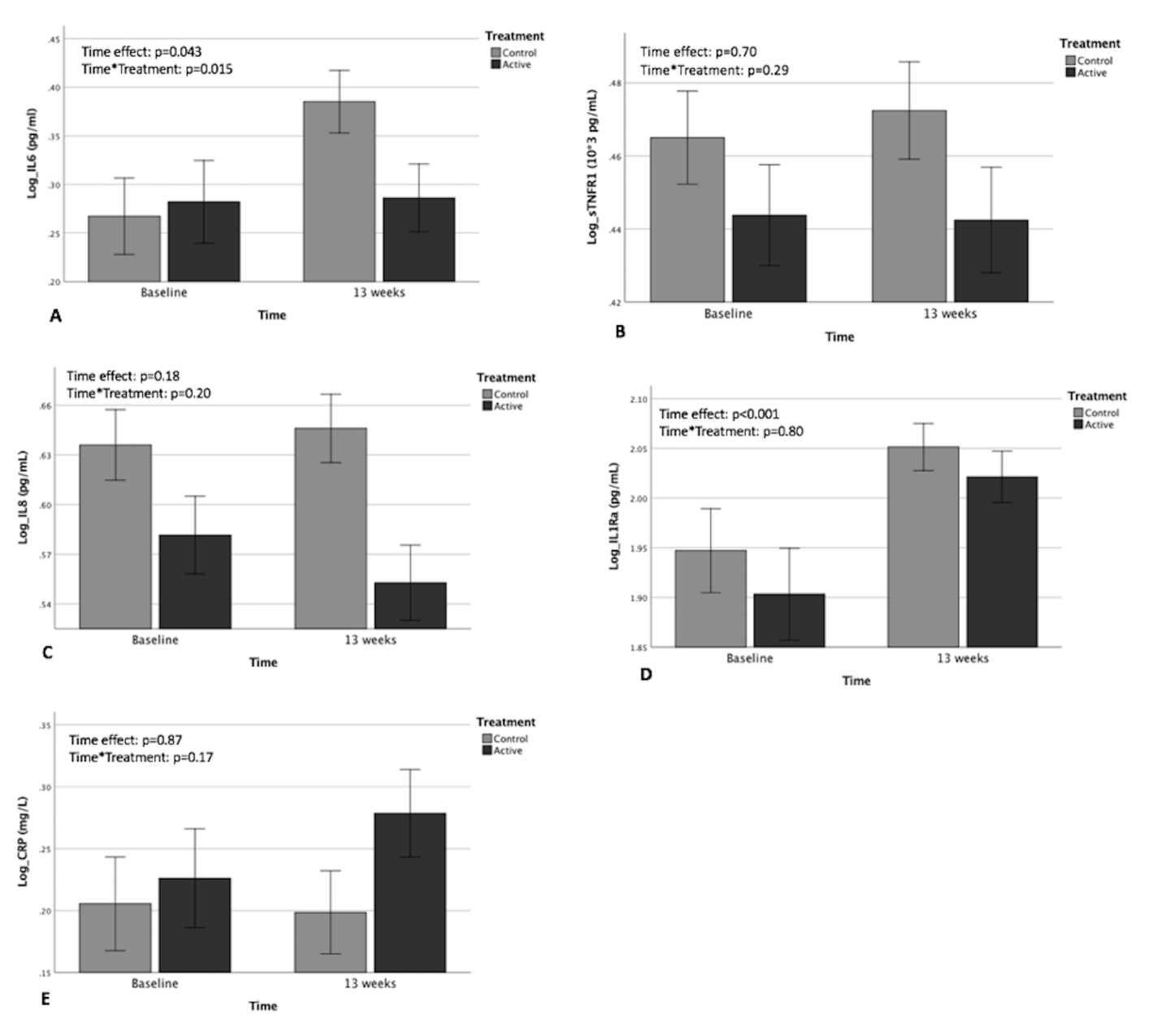


**Supplementary Figure 1.** **Effects of active versus control intervention on inflammatory markers in participants with CRP-levels <10mg/L.** A) IL-6 B) sTNFR1 C) IL-8 D) IL1Ra E) CRP Bars represent mean values ± 1SD corrected for mean dietary VitD intake as a covariate. Repeated measures ANCOVA.
